# Global Elimination of Lymphatic Filariasis: A “Mass Uprising of Compassion”

**DOI:** 10.1371/journal.pntd.0002264

**Published:** 2013-08-29

**Authors:** David G. Addiss

**Affiliations:** Task Force for Global Health, Decatur, Georgia, United States of America; Centers for Disease Control and Prevention, United States of America

## Introduction

Hospitals and medical centers often cite compassion as a core value in their mission statements. In contrast, the importance of compassion in global health is rarely acknowledged, even though it is a significant source of motivation and sustenance for those working in the field. The Global Programme to Eliminate Lymphatic Filariasis (GPELF) provides an illustrative example of the role and promise of compassion in global health. It was established in 1998 to alleviate and prevent immense human suffering caused by the neglected tropical disease (NTD) lymphatic filariasis (LF).

From its beginning, the GPELF was conceived as having two “pillars”: one to interrupt transmission of the parasites that cause LF and the other to care for those who already have LF-related disease [1]. Inclusion of a morbidity management pillar distinguished the GPELF from efforts to eradicate smallpox, polio, and Guinea worm disease, which focused primarily, if not exclusively, on interrupting transmission. This two-pronged approach was initially criticized on the basis that efforts to reduce the suffering of those affected would divert attention and resources from the principal goal of stopping LF transmission [2].

Despite these reservations, the GPELF adopted morbidity management for three main reasons. First, the World Health Assembly (WHA) resolution that launched the GPELF (WHA 50.29) called for eliminating LF “as a public health problem.” The public health problem in question was not microfilaremia, but rather the stigmatizing and disfiguring conditions of lymphedema, affecting some 15 million persons, and hydrocele, affecting some 25 million men. Second, it was thought that providing clinical care to those who already had LF-related disease could enhance the acceptability of preventive chemotherapy to interrupt transmission. Cantey and colleagues recently documented this beneficial effect in a study from Orissa, India [3]. Finally, and most importantly, relieving suffering through morbidity management was considered the right thing to do. Compassion demanded it.

## Compassion and the GPELF

Social psychology teaches that compassion is comprised of three key elements: cognitive awareness, emotional resonance, and compassionate action. The GPELF is characterized by all three. First, a compassionate response to suffering requires that one first be aware of its existence. In a speech to the Centers for Disease Control and Prevention (CDC) in 1984, Bill Foege said: “If we are to maintain the reputation this institution now enjoys, it will be because in everything we do, behind everything we say, as the basis for every program decision we make—we will be willing to see faces.” This was an extraordinary message for a major public health institution with responsibilities for the health of populations, not individuals. The CDC's reputation would depend not on programmatic effectiveness, measurable outcomes, or epidemiologic prowess, but on compassion—the willingness of its employees, collectively, to see the faces of suffering.

Many who established the GPELF had studied LF as scientists and they were acutely aware of the personal suffering, stigmatization, and disability that it caused. They had seen the faces—and could not forget them. Inspired by the pioneering clinical work of Dr. Gerusa Dreyer in Brazil [4] and Professor R.K. Shenoy in India [5], they insisted that priority be given to relieving LF-related suffering. During the past two decades, our collective awareness of the magnitude and nature of this suffering has grown tremendously, thanks to excellent studies by social scientists in Ghana, Brazil, Haiti, the Dominican Republic, India, and elsewhere (summarized in [6]).

Second, compassion requires emotional attunement or empathy—the ability to *feel* the suffering of the other. The LF program has engaged the emotions since it began. A 1997 booklet by the World Health Organization (WHO) that promoted the cause of LF elimination was punctuated with sentences from a letter by a Ghanaian woman with advanced LF-related lymphedema [7]. “Dear sir,” she begins, “I am writing with the hope that you can help me.” A few pages later, she continues: “I kneel and plead to be touched by your innermost heart for a humanitarian feeling, to try and do your best to help me.” Although the role of emotion as a motivating force, either individually or collectively, is rarely discussed, virtually everyone who is actively engaged in LF elimination has a story, a lived experience of human suffering that was deeply moving—and that lies at the core of their motivation for the work.

Third, compassion is characterized by action. The Fourteenth Dalai Lama said that compassion “is not just a wish to see sentient beings free from suffering, but an immediate need to intervene and actively engage, to try to help…A person who has attained stability in his or her compassion training…should now be out, running around like a mad dog, actively engaged in acts of compassion” [8]. By any standard, the action mobilized by the GPELF to eliminate LF-related suffering for future generations has been “mad dog” impressive. Some 3.9 billion preventive chemotherapy treatments have been delivered since 2001. The GPELF is one of the most rapidly upscaling programs in public health history, having engaged hundreds of thousands of workers and now treating more than 500 million persons each year [9].

## Are We Seeing the Faces?

Unfortunately, the morbidity management pillar, which WHO describes as “rooted in compassion” [10], has not fared as well. In 2011, 53 of 73 LF-endemic countries had preventive chemotherapy programs, while only 27 reported activities in morbidity management [10]. Several reasons likely contribute to this. Preventive chemotherapy has proven such a powerful intervention for interrupting LF transmission that most of the attention and resources in the GPELF have been devoted to scaling it up. Because of its success, preventive chemotherapy has become the overriding organizing principle not only for the elimination of LF, but also for the control of other NTDs [11], [12]. In addition, recommended procedures and guidelines for management of hydrocele and lymphedema in LF-endemic areas were not well-established when the GPELF began. Further, effective national programs to manage lymphedema and hydrocele require extensive collaboration with clinical health services, beyond the purview and experience of many LF program managers. A more nuanced and sophisticated matrix approach toward LF morbidity management is emerging that mobilizes surgical services to manage urogenital LF and integrates lymphedema management with clinical care for conditions such as diabetic foot, leprosy, and Buruli ulcer [13]. In many areas, this integrated approach, although not “solely owned” by the LF program, will be essential for the LF program to achieve its goals.

A more subtle and pervasive reason for the slow uptake of morbidity management in the GPELF lies in the corrosive forces that inhibit and obstruct compassion in many global health programs. Sustaining the empathic connection required for compassion—seeing the faces behind the numbers—is difficult when working to improve the health of hundreds of millions of people, across great geographic distances. What does it mean to have compassion for entire populations? The global scope of the LF program requires the collaboration of many complex organizations, often with competing agendas and historic rivalries. Motivations other than compassion, such as economic profit, political and military hegemony, and personal ambition, are notoriously active. Our collective silence on compassion in our work isolates us as individuals and allows these other forces to operate unchallenged.

## Addressing the Challenges

Much remains to be done if the 2020 target for LF elimination is to be met [10]. In addition to addressing the remaining technical, logistical, and financial challenges, it will be necessary to simultaneously attend to four pairs of activities, each of which holds the tension of paradox. First, we need to maintain focus on eliminating transmission *and* expand our peripheral vision to include, to a much greater extent, those with LF disease. The two pillars are complementary and mutually reinforcing, not conflicting. Second, extending the benefits of the GPELF to those who currently suffer from LF-related disease will require national LF elimination programs that are cohesive and unified *and* that engage different sectors of the health care system—a more sophisticated and collaborative approach. Third, we need to be able to see the faces *and* the numbers—and to do this *at the same time* [14]. Finally, we need to combine compassion for individuals with action at the population level.

How might we begin to see both the faces and the numbers, to combine compassion for individuals with action at the population level? We might start by sharing our stories, by venturing to speak more openly about the compassion that motivates our work and sustains our spirits. Many of us find that travel to the field, literally to “see the faces,” revitalizes our efforts to improve the health of populations. We keep photos in our workplace that remind us of individuals whose lives have touched us. As we break our collective silence on compassion, other possibilities and practices will undoubtedly emerge.

The late Steve Ben Israel, an American comedian, anarchist, and performance artist, said that the intent of his life's work was to “foment a mass uprising of compassion” [15]. While the GPELF has not fully extended the benefits it initially promised to those with LF-related disease, as a global response to human suffering, it represents a spectacular—if unfinished—mass uprising of compassion. By connecting more deeply with the compassionate impulse within us, by cultivating the capacity to be fully present to the faces of suffering, we will be better equipped, individually and collectively, to realize the GPELF's tremendous potential.

## References

[1] Seim A, Dreyer G, Addiss D (1999) Controlling morbidity and interrupting transmission: twin pillars of lymphatic filariasis elimination. Rev Soc Bras Med Trop 32: 325–328. 10380574 10.1590/s0037-86821999000300022

[2] Addiss DG (2010) Global elimination of lymphatic filariasis: addressing the public health problem. PLoS Negl Trop Dis 4: e741 doi:10.1371/journal.pntd.0000741 20614015 10.1371/journal.pntd.0000741 PMC2894130

[3] Cantey PT, Rout J, Rao G, Williamson J, Fox LM (2010) Increasing compliance with mass drug administration programs for lymphatic filariasis in India through education and lymphedema management programs. PLoS Negl Trop Dis 4: e728 doi:10.1371/journal.pntd.0000728 20628595 10.1371/journal.pntd.0000728 PMC2900179

[4] Dreyer G, Addiss D, Dreyer P, Noroes J (2002) Basic lymphoedema management: treatment and prevention of problems associated with lymphatic filariasis. Hollis, NH: Hollis Publishing Co. 112 pp.

[5] Shenoy RK (2008) Clinical and pathological aspects of filarial lymphedema and its management. Korean J Parasitol 46: 119–125. 18830049 10.3347/kjp.2008.46.3.119 PMC2553332

[6] Zeldenryk LM, Gray M, Speare R, Gordon S, Melrose W (2011) The emerging story of disability associated with lymphatic filariasis: a critical review. PLoS Negl Trop Dis 5: e1366 doi:10.1371/journal.pntd.0001366 22216361 10.1371/journal.pntd.0001366 PMC3246437

[7] WHO (1997) Lymphatic filariasis: elimination of a public health problem in our lifetime. Geneva: World Health Organization. Report Number WHO/CTD/FIL/97.4. 20 pp.

[8] Davidson RJ, Harrington A (2002) Visions of compassion: western scientists and Tibetan Buddhists examine human nature. New York: Oxford University Press. 263 pp.

[9] WHO (2012) Global programme to eliminate lymphatic filariasis: progress report, 2011. Wkly Epidemiol Rec 87: 345–356. 22977953

[10] WHO (2010) Progress report 2000–2009 and strategic plan 2010–2020 of the global programme to eliminate lymphatic filariasis: halfway towards eliminating lymphatic filariasis. Geneva: World Health Organization.

[11] Hotez PJ, Molyneux DH, Fenwick A, Kumaresan J, Ehrlich Sachs S, et al (2007) Control of neglected tropical diseases. N Engl J Med 357: 1018–1027. 17804846 10.1056/NEJMra064142

[12] Utzinger J, Raso G, Brooker S, De Savigny D, Tanner M, et al (2009) Schistosomiasis and neglected tropical diseases: towards integrated and sustainable control and a word of caution. Parasitology 136: 1859–1874. 19906318 10.1017/S0031182009991600 PMC2791839

[13] WHO (2012) Managing morbidity and preventing disability in the Global Programme to Eliminate Lymphatic Filariasis: WHO position statement. Geneva: World Health Organization. Report Number WHO/HTM/NTD/PCT/2011.8. 22191103

[14] Foege B, Rosenberg M (1999) Public and community health. In: Gilkey RW, editor. The 21^st^ century health care leader. San Francisco: Josey-Bass. pp. 85–90.

[15] Vitello P (2012 June 17) Steve Ben Israel, 74, a living theater performance artist. The New York Times; A22.

